# Fatal Calf Pneumonia Outbreaks in Italian Dairy Herds Involving *Mycoplasma bovis* and Other Agents of BRD *Complex*

**DOI:** 10.3389/fvets.2021.742785

**Published:** 2021-09-10

**Authors:** Angela Fanelli, Margie Cirilli, Maria Stella Lucente, Aya Attia Koraney Zarea, Domenico Buonavoglia, Maria Tempesta, Grazia Greco

**Affiliations:** ^1^Department of Veterinary Medicine, University of Bari Aldo Moro, Bari, Italy; ^2^Department of Microbiology and Immunology, National Research Centre, Cairo, Egypt

**Keywords:** *Mycoplasma bovis*, bovine respiratory disease (BRD), fatal pneumonia, seasonal-trend decomposition based on loess (STL), Italy

## Abstract

*Mycoplasma bovis* is increasingly recognized worldwide as an important cause of disease with major welfare and production impairments on cattle rearing. Although it was detected in veal calves and beef cattle, little is known on the infection impact and on its temporal morbidity pattern in Italian dairy herds. Thus, this study aimed to investigate the involvement of *M. bovis* on fatal calf pneumonia outbreaks that occurred during 2009–2019 in 64 Italian dairy farms. Furthermore, a deeper diagnostic workup of concurrent infection with other viral and bacterial respiratory pathogens was assessed. Out of the investigated fatal pneumonia cases, *M. bovis* was frequently detected (animal prevalence, 16.16%; 95%CI, 11.82–21.33; herd prevalence, 26.56; 95%CI, 16.29–39.08) either as the single agent of the disease in more than half of the positive samples (20/37) or in concurrent infections with *Histophilus somni* (9/37, 24.3%), *Mannheimia haemolytica* (6/37, 16.621%), *Trueperella pyogenes* (1/37, 2.70%), *Pasteurella multocida* (1/37, 2.70%), bovine respiratory syncytial virus (5/37, 13.51%), and bovine viral diarrhea virus (2/37, 5.55%). Based on time-series analysis, *M. bovis* was recorded in the area since 2009 with outbreaks displaying a clear morbidity seasonal pattern with peaks in April (43.21%) and in September (13.51%). This might be due to the stressing conditions during spring and late summer periods. Results of this study highlight that *M. bovis* infection warrants consideration, and control measures are needed given its involvement in lethal pneumonia outbreaks in dairy herds from an extended area.

## Introduction

*Mycoplasma bovis* is a cell-wall-less bacterial pathogen, included in the class of *Mollicutes* (1). It is recognized as a causative agent of several diseases in cattle that have severe economic consequences for producers (1). In dairy cattle, *M. bovis* is probably the most common causative agent of mycoplasma mastitis, with arthritis and otitis media sometimes observed in infected animals (2). Noteworthy, it contributes to the occurrence of the bovine respiratory disease complex (BRD), a multifactorial syndrome consisting of chronic bronchopneumonia and pharyngitis, although often the infection can remain subclinical (3).

Under natural condition, *M. bovis* interanimal transmission occurs mainly via colostrum, milk, air-borne, intrauterine, and contaminated semen (4). Moreover, *M. bovis* has the capability to produce a biofilm, making it possible for the bacterium to survive in the environment while withstanding the action of heat or desiccation (5). It was shown that *M. bovis* is able to maintain viability in the environment for months at low temperatures and weeks at room temperature on a variety of substrates in infected herds (5).

After infection, *M. bovis* can spread through the bloodstream, establishing a long-term persistent infection through escaping the immune response. *M. bovis*, as primary agent or under the action of concomitant stressing factors such as weaning, transport, or relocation to feedlots, may impair the host immune system efficiency resulting in the onset of the disease (6) including severe, often fatal, pneumonia (7, 8). Case fatality is estimated to be 5–10% or higher in more severe cases, with morbidity reaching 35% (3).

Although the real economic burden of the disease has not yet been evaluated, in the last few years, *M. bovis* has been increasingly recognized as a significant concern for the cattle industry due to milk loss, decreased weight gain, and cost for veterinary and drug treatments. Furthermore, *M. bovis* infection is particularly important from an animal welfare perspective being the persistent debilitating disease that is unresponsive to therapy (9). Indeed, the antibiotic resistance, the presence of asymptomatic carriers, and the lack of effective vaccines are identified as the major constraints in the control of the disease (10).

*M. bovis* was included in the EU-funded DISCONTOOLS project (https://www.discontools.eu/), which provides a decision tool for diseases prioritization in order to improve the application of preventive and control measures (11). According to epidemiological investigations, the most effective way to prevent the infection spread is based on the strict adoption of biosecurity measures and restrictions on animal movements (12). Currently, although in Europe, *M. bovis* occurrence does not incur official restrictions on livestock trade, nevertheless, some importing countries are requesting cattle to test free from the infection (10).

In Italy, *M. bovis* was detected in veal calves and beef cattle with pneumonic lesions at slaughter (13) and in batches of imported bulls stabled in farms in northern Italy (14). The infection was also recently reported in healthy and BRD symptomatic beef cattle imported from France in Southern Italy (15). Nonetheless, information on *M. bovis* infection in dairy herds is scarce.

In this study, we document the involvement of *M. bovis* in fatal calf pneumonia outbreaks in Italian dairy herds together with its spatial–temporal distribution. Additionally, concurrent infections with other viral and bacterial respiratory pathogens were recorded.

## Materials and Methods

From 2009 to 2019, 229 lung samples of calves (<11 months) from 64 dairy farms experiencing outbreaks of fatal calf pneumonia were submitted for postmortem diagnostic workup to the Laboratory of Infectious Diseases of the Department of Veterinary Medicine of the University of Bari (Italy). The farms were from Apulia, Basilicata, Campania, and Calabria regions and managed under either semi-intensive production systems farming, characterized by access to pasture grazing during certain periods of the year or intensive farming, in case of larger herds, where animals were housed in a free stabling system.

*M. bovis* and a panel of concurrent infectious agents involved in BRD were investigated. In detail, bacteria and viruses including *Histophilus somni, Mannheimia haemolytica, Pasteurella multocida, Trueperella pyogenes*, bovine herpesvirus-1 (BoHV-1), bovine viral diarrhea virus (BVDV), bovine respiratory syncytial virus (BRSV), and bovine coronavirus (BCoV) were included. Samples were submitted for DNA and RNA extraction using commercial kits (Qiagen, Milan, Italy) and subsequently analyzed for the infectious agents by using quantitative PCR (qPCR) or conventional PCR (cPCR) assays as already described (Supplementary Table S1). For each pathogen, prevalence was computed at animal (AP) and herd (HP) levels along with the 95% confidence interval (95%CI). The spatial distribution of farms that tested positive for *M. bovis* was mapped by using QGIS software version 3.6.0 (16). To respect farmers' privacy, herds were represented at municipality level. A heatmap was built to better visualize the case distribution. The analysis of pathogen species co-occurrence was performed to detect pairs of pathogens that infect hosts more or less frequently than expected. A probabilistic model, as developed by Veech (17), was used to test for pairwise patterns of species co-occurrence, with the significance level α, set at ≤ 0.05.

With the aim of providing valuable insights on disease patterns, time-series analysis was performed, which is being widely implemented in the field of epidemiology (18–21). For the purpose of this study, an outbreak was defined as one of more cases occurring in the same epidemiological unit and month. The number of *M. bovis* outbreaks by calendar month was formatted into time series, and a seasonal trend decomposition based on loess (STL) was used to identify relevant seasonal patterns. Records from 2019 were excluded in the temporal analysis, as data for some months was missing. To extract the seasonality, the loess window was assigned to 13, as it is recommended to use the next odd number following the number of observations in each seasonal cycle (22). Scale bars were included in the plots to describe the range of each component of the decomposition. The relative interquartile range (IQR) was used to measure the variability in the data explained by each component. The relative IQR is computed as the IQR of each component of the decomposition compared to the IQR of the raw data. Quantile plot of the residuals was drawn to ensure that they approximate a normal distribution. All the statistics were done using R software 3.5.2 (23).

## Results

**Figure 1:**
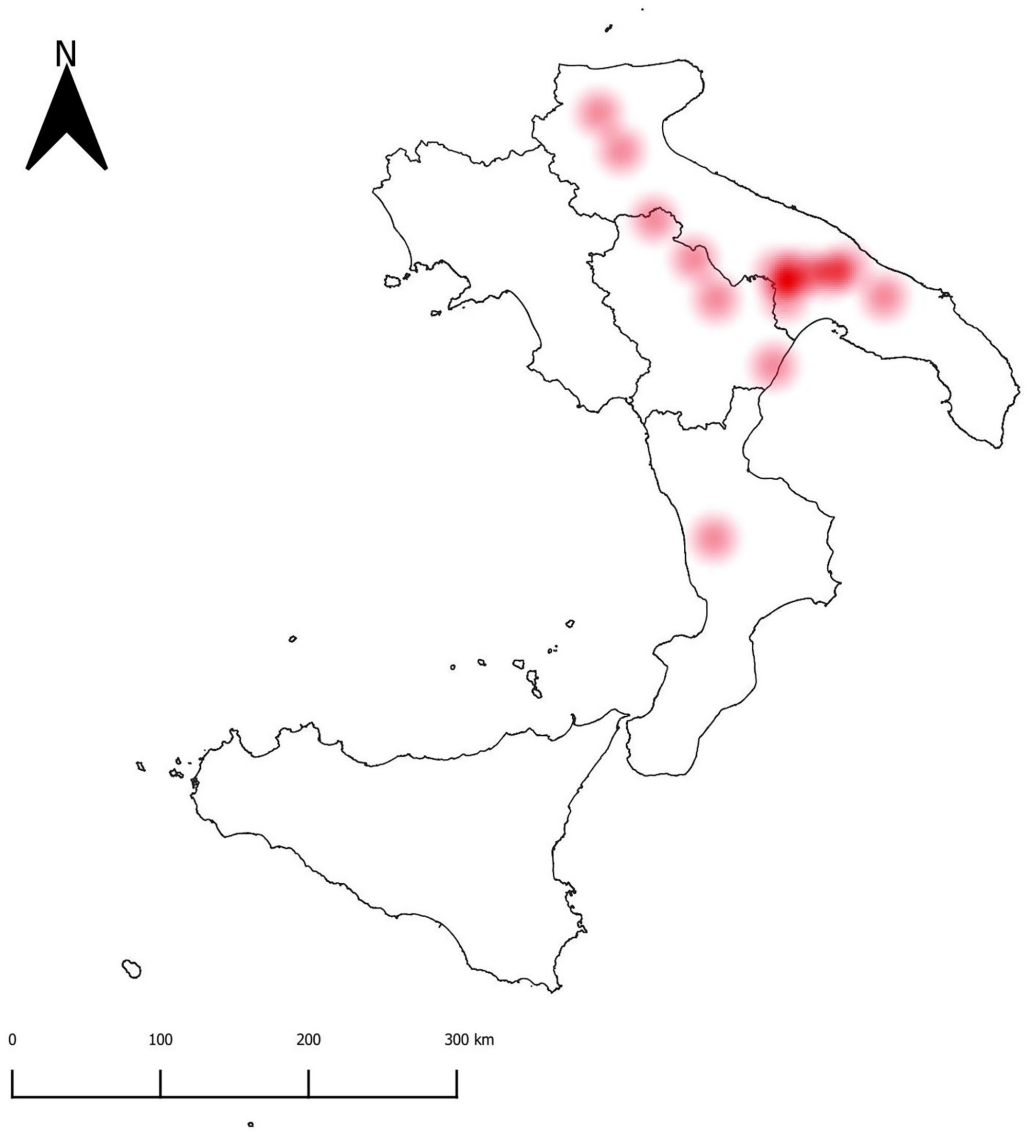
Heatmap showing the density of *M. bovis* positive herds.

The prevalence values at animal (AP) and herd (HP) level for all the detected bacteria and viruses are displayed in Table 1. *M. bovis* was the most frequently detected pathogen in the study area (AP, 16.16%; 95%CI, 11.82–21.33; HP, 26.56%; 95%CI, 16.29%−39.08%) (Figure 1). Furthermore, *M. bovis* was detected as single pathogen in more than half of the positive lung samples (20/37). Details of the coinfections (*n* = 17) are provided in Supplementary Table S2. In particular, *M. bovis* was detected with *P. multocida* (*n* = 1, 2.70%), *M. haemolytica* (*n* = 6, 16.21%), *H. somni* (*n* = 9, 24.3%), and *T. pyogenes (n* = 1, 2.70%), and BVDV (*n* = 2, 5.54%), and BRSV (*n* = 5, 13.51%). No mixed infection of *M. bovis* with BCoV or BoHV-1 was recorded.

**Table 1:** Prevalence at animal and herd levels for *Mycoplasma bovis* and other pathogens associated with fatal pneumonia in farms from Southern Italy.

**Pathogen**	**Animal prevalence (%) (95%CI)**	**Herd prevalence (%) (95%CI)**
*M. bovis*	16.16 (11.82–21.33)	26.56 (16.29–39.08)
*P. multocida*	4.80 (2.42–8.43)	6.25 (1.72–15.23)
*M. haemolytica*	13.53 (9.38–18.66)	20.31 (11.28–32.22)
*H. somni*	7.86 (4.72–12.13)	7.81 (2.58–17.29)
*T. pyogenes*	1.31 (0.03–3.78)	3.12 (0.38–10.87)
BoHV-1	4.80 (2.42–8.43)	12.50 (5.55–23.15)
BVDV	9.17 (5.76–13.67)	12.50 (5.55–23.15)
BRSV	6.98 (4.04–11.09)	9.37 (3.51–19.29)
BCoV	11.79 (7.91–16.68)	3.12 (0.38–10.83)

*BoHV-1, bovine herpesvirus-1; BVDV, bovine viral diarrhea virus; BRSV, bovine respiratory syncytial virus; BCoV, bovine coronavirus*.

**Figure 2:**
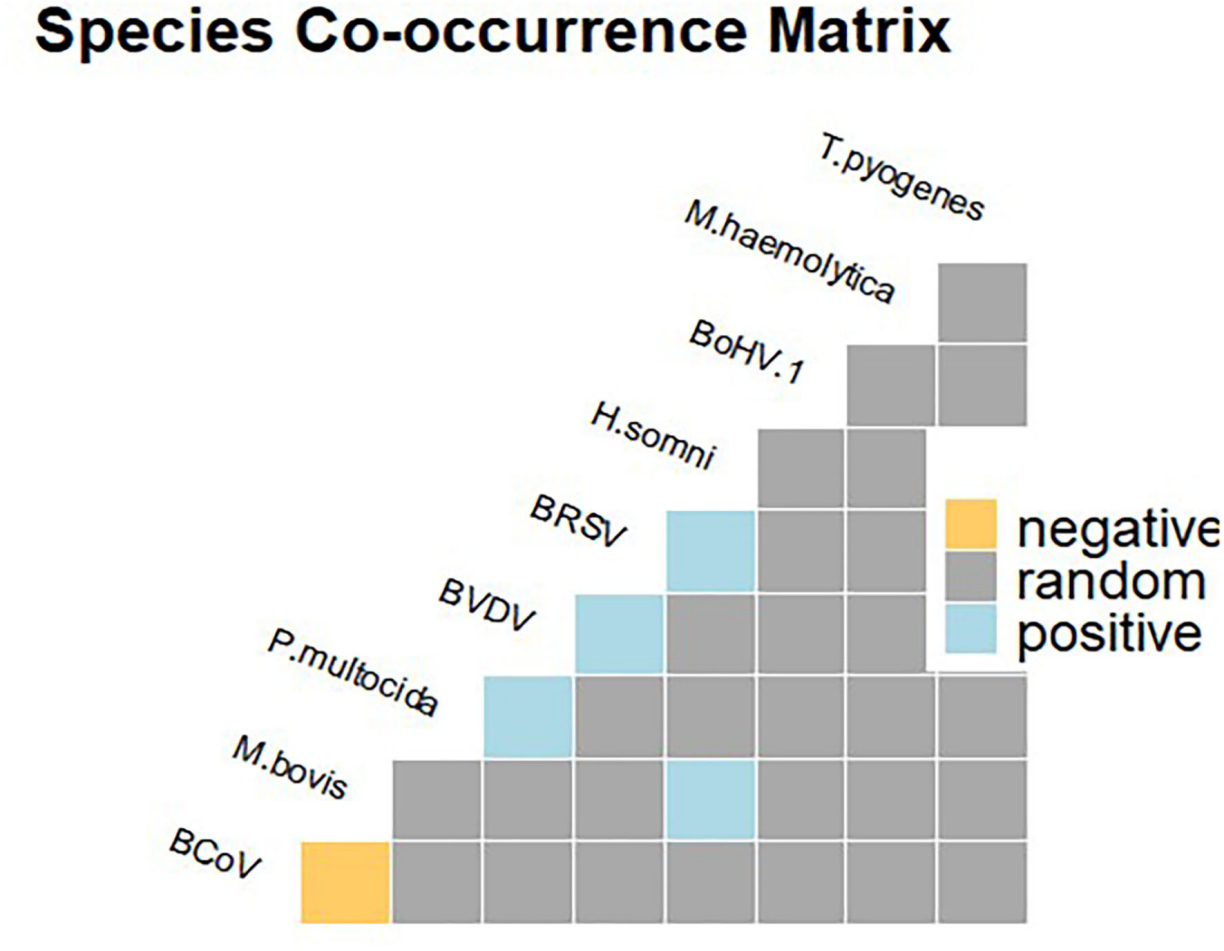
Species co-occurrence matrix showing positive (in blue), negative (in yellow), and random (gray) associations.

Out of 36 potential species pairs (Figure 2), 23 were considered in the co-occurrence analysis after removing the other 13 (36.11 %) with the expected outcomes lower than one. The majority of the pathogen pairs had random associations, while non-random associations were recorded for a few pairs including *M. bovis–H. somni, P. multocida–*BVDV, BVDV–BRSV, and BRSV–*H. somini* (Figure 2). Furthermore, a negative significant association was detected for the *M. bovis* and BCoV pair, as these two species co-occurred at a frequency less than expected.

**Figure 3:**
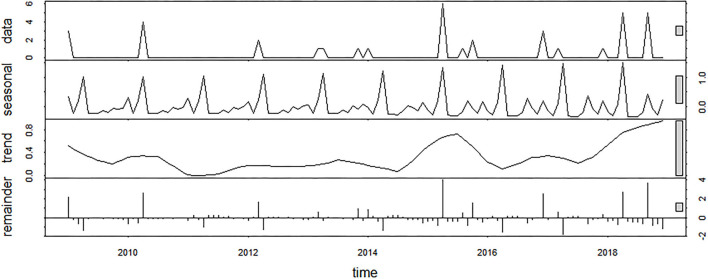
Seasonal decomposition of *M. bovis* outbreaks using an additive model (raw data, seasonal component, trend component, and remainder).

From 2009 to 2018, 37 outbreaks associated with *M. bovis* infection were recorded, with the majority occurring in 2015 (*n* = 6) and 2018 (*n* = 5). Considering the STL decomposition, the scale bars included in the plots showed that the remainder component of the decomposition had the highest IQR (Figure 3). This is mainly due to the considerable peak of outbreaks observed in 2015 and 2018, for which trend and seasonality components were not able to explain the variability of the data. The relative IQR measures, which exclude extreme values, were 37.66% for the seasonal component, 19.23% for the trend component, and 40.59% for the remainder. The seasonality pattern was constant over the period of analysis. Out of 37 *M. bovis* outbreaks, 16 (43.21%) occurred in April, 5 (13.51%) in September, and 4 (10.81%) in December, with only sporadic events spread over other months (i.e., January, March, October, or November) (Supplementary Figure S1).

## Discussion

In this study, *M. bovis* was the agent most frequently detected from fatal calf pneumonia cases (AP, 16.16%; 95%CI, 11.82–21.33) occurring in dairy farms (HP, 26.56; 95%CI, 16.29–39.08) from Southern Italy during a 10-year period. *M. bovis* was detected as the single agent in half of positive lungs or in association with *H. somni* (9/37, 24.3%), followed by *M. haemolytica* (6/37, 16.21%), *T. pyogenes* (1/37, 2.70%), *P. multocida* (1/37, 2.70%), BRSV (5/37, 13.51%), and BVDV (2/37, 5.54%) (Figure 2). However, as the samples all came from fatal cases, it was not possible to determine how prevalent this pathogen is in non-fatal cases.

*M. bovis* occurrence has been described worldwide with an increasing number of reports of calf pneumonia cases from Britain (24), Ireland (25), Canada (26, 27), and Switzerland (28). In Italy, *M. bovis*, together with other BRD agents, was recently reported both in healthy and sick animals (14, 15, 29), stressing the importance of the agent as a primary concern in the Italian feedlot system. To date, no studies investigated fatal pneumonia outbreaks involving *M. bovis* with regard their temporal pattern. Thus, this work provides an important contribution to the knowledge on *M. bovis*, representing the first report investigating a series of fatal calf pneumonia outbreaks that occurred in dairy herds during a 10-year period in Southern Italy.

It is well-known that BRD is a multifactorial syndrome involving different bacterial or viral infectious agents and management factors, thus posing a real challenge in the diagnose without laboratory investigations (28, 30). As reported in previous studies, *M. bovis* was found to be positively associated with BVDV infection (Figure 2), although at low frequency, thus supporting for the synergistic role of the two pathogens in the occurrence of fatal bronchopneumonia (27, 31). Furthermore, there was evidence for a significant association between co-occurrence of *M. bovis* and *H. somni* and fatal pneumonia cases. This finding is of particular interest, *H. somni* being an emerging pathogen in the study area (15). Furthermore, *H. somni* was also shown to be widely implicated in pneumonia cases of cattle under 12 months of age in Ireland (32). Finally, in line with previous studies, coinfection of *M. bovis* and *M. haemolytica* was found (33), although the probabilistic model analysis suggests that this was a random association.

The main objective of the study was to investigate the occurrence of *M. bovis* in fatal calf pneumonia cases. Nevertheless, this work also highlighted the spread of other viral and bacterial respiratory pathogens in Italian dairy herds. Indeed, in line with previous studies, significant associations between the pairs of pathogens, including BVDV–BRSV (34) and BRSV–*H. somni* (35), were recorded. These pairs occurred at a frequency higher than expected, hinting that different distinct synergism of pairwise pathogens may occur in BRD pathogenesis.

Based on STL analysis, the occurrence of the *M. bovis* outbreaks revealed a time-dependent behavior with peaks in April (43.21%) and September (13.51%) (Figure 3). This is of particular importance, as detecting the relevant risk periods provides useful information for disease preparedness. Nevertheless, the seasonal pattern described in this work did not overlap those ones reported in previous studies performed in indoor systems that recorded the highest incidence of pneumonia during colder seasons (14). Possibly, harsh temperature fluctuations rather than the cold temperatures may have played a role in the outbreak occurrence due to the remarkable changes in weather condition experienced during springtime and late summer in our study area. Given the seasonal importance, the effect of climatic stress on the hosts' disease susceptibility should be better investigated with further research.

Based on the time-series analysis, *M. bovis* occurrence was recorded in the area since 2009 (Figures 2, 3), but the sources for the outbreaks could not be identified, although the putative role of subclinical infected livestock introduced via the trade cannot be ruled out. Indeed, previous studies documented the introduction of the common BRD pathogens via imported cattle from France (15). On the other hand, other studies identified the presence of *M. bovis* infection in subclinical adult animals as the potential source of infection to the newly imported calves (14).

To conclude, based on the time-series analysis, *M. bovis* was in the area since 2009, with outbreaks displaying a clear morbidity seasonal pattern with peaks in April (43.21%) and in September (13.51%), which might be due to unknown stress conditions during spring and late summer periods. Results of this study highlight that *M. bovis* infection warrants consideration, and control measures are needed given its involvement in lethal pneumonia outbreaks in dairy herds from an extended area. One limitation of the present study is the lack of anamnestic data on the infected animals. Additionally, samples were not tested for bovine adenovirus (BAdV) and bovine parainfluenza virus 3 (BPIV-3), which have been recently found in BRD-affected beef steers in Southern Italy (36). Despite these limitations, results from this study made an important contribution from the animal health perspective.

## Data Availability Statement

The raw data supporting the conclusions of this article will be made available by the authors, without undue reservation.

## Ethics Statement

Ethical review and approval was not required for the animal study because The study included samples that were voluntary submitted from the animal owners to the diagnostic laboratory for diagnostic workup. Written informed consent was obtained from the owners for the participation of their animals in this study.

## Author Contributions

GG: conceptualization, data curation and analysis, methodology, investigation and supervision, and writing. AF: data curation, formal analysis, and writing—original draft preparation. MC and ML: laboratory analysis. AF, GG, MC, ML, AZ, DB, and MT: reviewing and editing. All authors contributed to the article and approved the submitted version.

## Conflict of Interest

The authors declare that the research was conducted in the absence of any commercial or financial relationships that could be construed as a potential conflict of interest.

## Publisher's Note

All claims expressed in this article are solely those of the authors and do not necessarily represent those of their affiliated organizations, or those of the publisher, the editors and the reviewers. Any product that may be evaluated in this article, or claim that may be made by its manufacturer, is not guaranteed or endorsed by the publisher.
