# Supplementary material for: Fatal Calf Pneumonia Outbreaks in Italian Dairy Herds Involving Mycoplasma bovis and Other Agents of BRD Complex
Source: Front Vet Sci. 2021 Sep 10;8:742785. doi: 10.3389/fvets.2021.742785 (PMC8462733; doi:10.3389/fvets.2021.742785)
Supplement: Supplementary file 1 [file Table_1.docx]

Supplementary Material

**Table S1.** Target and primer/probe sets used in this study.

| *Target* | Target gene | Primer name | bp | References |
| --- | --- | --- | --- | --- |
| *M. bovis* | *opp*D | PMB996-F: 5**’**-TCAAGGAACCCCACCAGAT-3’ | 71 | 1 |
|  |  | PMB1066-R: 5**’**-AGGCAAAGTCATTTCTAGGTGCAA-3**’** |  |  |
|  |  | probe Mbovis1016: FAM-TGGCAAACTTACCTATCGGTGACCCT-TAMRA |  |  |
| *M. haemolytica* | *lkt*A-*art*J intergenic region | Mh-F: 5’-GTCCCTGTGTTTTCATTATAAG-3’ | 385 | 2 |
|  |  | Mh-R: 5’-CACTCGATAATTATTCTAAATTAG-3’ |  |  |
| *P. multocida* | KMT1 | KMT1SP6-F: 5’-GCTGTAAACGAACTCGCCAC-3’ | 460 | 3 |
|  |  | KMT1T7-R: 5’-ATCCGCTATTTACCCAGTGG-3’ |  |  |
| *H. somni* | 16S | HS453-F: 5’-GAAGGCGATTAGTTTAAGAG-3’ | 408 | 4 |
|  |  | HS860-R: 5’-TTCGGGCACCAAGTRTTCA-3’ |  |  |
| *T. pyogenes* | *plo* | PLO-F: 5’-CAGTCAAGGGTGAGTCTATT-3’ | 773 | 5 |
|  |  | PLO-R: 5’- CTTGAACTCTGTGGAAA-3’ |  |  |
| Bovine herpesvirus 1 | gE | F: 5’-CAATAACAGCGTAGACCTGGTC-3’ | 85 | 6 |
|  |  | R: 5’-GCTGTAGTCCCAAGCTTCCAC-3’ |  |  |
|  |  | Probe: 5’-FAM-TGCGGCCTCCGGGCTTTACGTCT-TAMRA |  |  |
| Bovine viral diarrheal virus | 5′UTR | F: 5’-GGGNAGTCGTCARTGGTTCG-3’ | 190 | 7 |
|  |  | R: 5’-GTGCCATGTACAGCAGAGWTTTT-3’ |  |  |
|  |  | P: 5’-FAM-CCAYGTGGACGAGGGCAYGC-TAMRA |  |  |
| Bovine coronavirus | Nucleocapsid | F: 5’-GGACCCAAGTAGCGATGAG-3’ | 90 | 8 |
|  |  | R: 5’-GACCTTCCTGAGCCTTCAATA-3’ |  |  |
|  |  | P: 5’-FAM-ATTCCGACTAGGTTTCCGCCTGG-TAMRA |  |  |
| Bovine respiratory syncytial virus | Nucleocapsid | F GCAATGCTGCAGGACTAGGTATAAT-3’ | 124 | 9 |
|  |  | R ACACTGTAATTGATGACCCCATTCT-3’ |  |  |
|  |  | P FAM-ACCAAGACTTGTATGATGCTGCCAAAGCA-TAMRA |  |  |

**References**

1- Sachse, K., Salam, H. S. H., Diller, R., Schubert, E., Hoffmann, B. and Hotzel, H. 2010. Use of a novel real-time PCR technique to monitor and quantitate Mycoplasma bovis infection in cattle herds with mastitis and respiratory disease. *Vet. J.* **186**: 299–303.

2-Angen O, et al. Respiratory disease in calves: microbiological investigations on trans-tracheally aspirated bronchoalveolar fluid and acute phase protein response. Vet Microbiol 2009;137:165-171.

3- Townsend KM, et al. Development of PCR assays for species- and type-specific identification of *Pasteurella multocida* isolates. J Clin Microbiol 1998;36:1096-1100.

4- Angen O, et al. Development of a PCR test for identification of *Haemophilus somnus* in pure and mixed cultures. Vet Microbiol 1998;63:39-48.

5-Zhang W, Liu X, Liu M, Ma B, Xu L, Wang J. Development of a multiplex PCR for simultaneous detection of Pasteurella multocida, Mannheimia haemolytica and Trueperella pyogenes. Acta Vet Hung. 2017 Sep;65(3):327-339. doi: 10.1556/004.2017.032. PMID: 28956490.

6- Wernike, K., Hoffmann, B., Kalthoff, D., König, P. and Beer, M. 2011. Development and validation of a triplex real-time PCR assay for the rapid detection and differentiation of wild-type and glycoprotein E-deleted vaccine strains of Bovine herpesvirus type 1. *J. Virol. Methods* **174**: 77–84.

7 -Mahlum, C. E., Haugerud, S., Shivers, J. L., Rossow, K. D., Goyal, S. M., Collins, J. E. and Faaberg, K. S. 2002. Detection of bovine viral diarrhea virus by TaqMan reverse transcription polymerase chain reaction. *J. Vet. Diagn. Invest.* **14**: 120–125.

8- Kishimoto M, Tsuchiaka S, Rahpaya SS, et al. Development of a one-run real-time PCR detection system for pathogens associated with bovine respiratory disease complex. *J Vet Med Sci*. 2017;79(3):517-523. doi:10.1292/jvms.16-0489

9- Boxus, M., Letellier, C. and Kerkhofs, P. 2005. Real Time RT-PCR for the detection and quantitation of bovine respiratory syncytial virus. *J. Virol. Methods* **125**: 125–130.
