# Supplementary material for: Fatal Calf Pneumonia Outbreaks in Italian Dairy Herds Involving Mycoplasma bovis and Other Agents of BRD Complex
Source: Front Vet Sci. 2021 Sep 10;8:742785. doi: 10.3389/fvets.2021.742785 (PMC8462733; doi:10.3389/fvets.2021.742785)
Supplement: Supplementary file 2 [file Table_2.docx]

Supplementary Material

**Table S2** Coinfections with *M. bovis*. The frequency of occurrence was computed over the total number of *M. bovis* positive samples (n=37)

| **Pathogens** | **Frequency** |
| --- | --- |
| *M. bovis* + *H. somni* | 5/37 |
| *M. bovis* + BRSV | 1/37 |
| *M. bovis* + *M.* *haemolytica* | 3/37 |
| *M. bovis* + *T. pyogenes* | 1/37 |
| *M. bovis* + *P. multocida* | 1/37 |
| *M. bovis+* BVDV*+ H. somni* | 1/37 |
| *M. bovis+* BRSV*+ H. somni* | 1/37 |
| *M. bovis+* BRSV*+ P. multocida* | 1/37 |
| *M. bovis+* BVDV+ *M.* *haemolytica* | 1/37 |
| *M. bovis+* BRSV*+ H. somni+ M.* *haemolytica* | 2/37 |
