# Supplementary figures and images for: Fatal Calf Pneumonia Outbreaks in Italian Dairy Herds Involving Mycoplasma bovis and Other Agents of BRD Complex
Source: Front Vet Sci. 2021 Sep 10;8:742785. doi: 10.3389/fvets.2021.742785 (PMC8462733; doi:10.3389/fvets.2021.742785)

## *Supplementary Material*

**Supplementary figure S1.** Seasonality extracted by year (from 2009 to 2018)

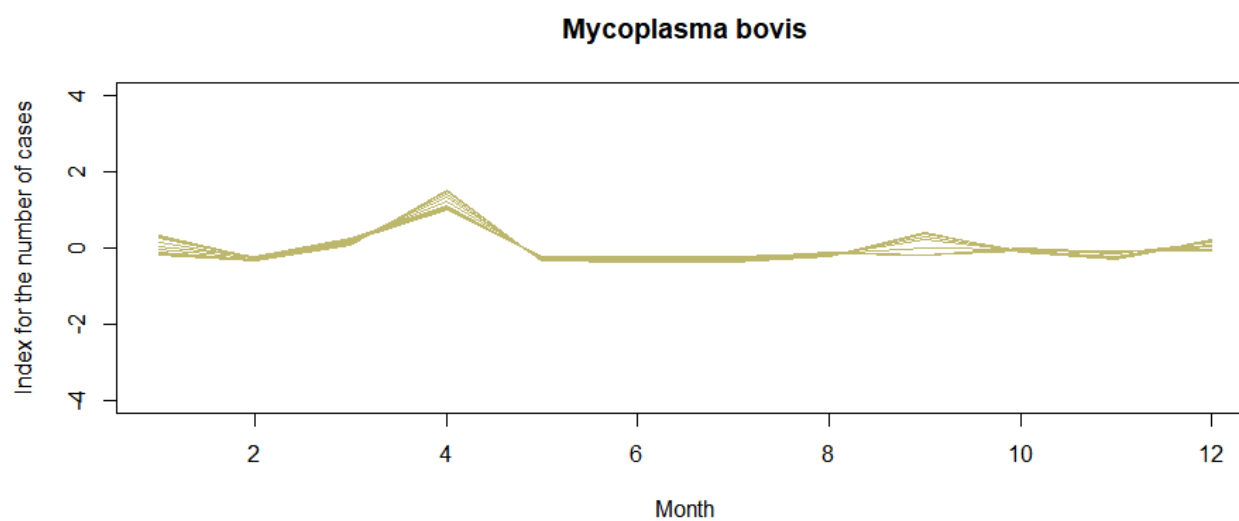

Supplement: Supplementary file 3 [file Image_1.pdf]
